# Mental disorders in childhood or adolescence and subsequent comprehensive school achievement

**DOI:** 10.1111/jcpp.70181

**Published:** 2026-06-04

**Authors:** Yan Li, Ripsa Niemi, Mai Gutvilig, Marko Elovainio, Marjo Flykt, Christian Hakulinen

**Affiliations:** ^1^ Department of Psychology, Faculty of Medicine University of Helsinki Helsinki Finland; ^2^ Finnish Institute for Health and Welfare Helsinki Finland

**Keywords:** Mental disorders, school achievement, age at onset, gender, register‐based data

## Abstract

**Background:**

Childhood mental disorders are associated with poorer school achievement, with potential negative long‐term socio‐economic consequences, but few large‐scale studies have examined this association, particularly the role of age at onset. Using a nationwide Finnish cohort, we examined the associations between mental disorders and school achievement, focusing on gender and age at onset.

**Methods:**

This nationwide cohort study used data from Finnish national registers. The study population consisted of all individuals born between January 1, 1990, and December 31, 2003, who completed their comprehensive education in Finland during the years 2005 to 2019 (*n* = 837,546). The exposure was diagnosis of any mental disorder or specific mental disorders between the age of 1 year and graduation. School achievement was measured using the grade point average (GPA), a summative measure of performance across all subjects during the final three years of comprehensive school.

**Results:**

Of the total study population (411,054 females, 426,492 males), 137,427 (16.41%) were diagnosed with a mental disorder before completing comprehensive school. Students with a mental disorder had lower school achievement compared with their peers without a diagnosis, −0.41 [95% CI, −0.41 to −0.40]. The association was stronger for female students, −0.45 [95% CI, −0.46 to −0.45], than for male students, −0.37 [95% CI, −0.38 to −0.37]. Later‐onset mental disorders were associated with poorer school achievement, a pattern observed across most specific mental disorders.

**Conclusions:**

Childhood mental disorders are linked to lower school achievement, particularly among females and those diagnosed at a later age, underscoring the need for targeted psychological support for females and adolescents.

## Introduction

Childhood is a critical period for mental and emotional development (Malik & Marwaha, 2023), and mental disorders during childhood can have a significant impact on long‐term educational achievements (Askeland et al., 2022; Lawrence, Dawson, Houghton, Goodsell, & Sawyer, 2019; Perry, Dunne, McFadden, & Campbell, 2008; Ringbom et al., 2022). Mental disorders diagnosed in childhood and adolescence, including psychotic disorders (e.g. schizophrenia) (Dickson et al., 2020; Glahn, Bearden, Bowden, & Soares, 2006), internalizing disorders (e.g. depression) (Esch et al., 2014; Maurizi, Grogan‐Kaylor, Granillo, & Delva, 2013; Pagerols et al., 2022), externalizing disorders (e.g. conduct disorder) (Galéra, Melchior, Chastang, Bouvard, & Fombonne, 2009; Liu, Huang, Kao, & Gau, 2017; McLeod, Uemura, & Rohrman, 2012; Sayal, Washbrook, & Propper, 2015), and neurodevelopmental conditions (e.g. autism spectrum disorder) (Galéra et al., 2009; McLeod et al., 2012; Sayal et al., 2015), have been associated with lower school achievement in adolescence. Children with mental disorders also exhibit significantly reduced school attendance rates (Lawrence et al., 2019), have a heightened risk for school expulsion (Perry et al., 2008) and suspension (Kang‐Yi et al., 2018), are more likely to drop out of high school (Askeland et al., 2022; Breslau, Lane, Sampson, & Kessler, 2008), and often experience delayed educational progression (Tempelaar et al., 2014).

Most prior studies on childhood mental disorders and school achievement have used small samples, retrospective designs, and focused on single‐disorder effects (Galéra et al., 2009; Glahn et al., 2006; Maurizi et al., 2013; Pagerols et al., 2022). To our knowledge, only two large‐scale studies on the topic have been conducted. First, a Danish registry study found that most mental disorders diagnosed before age 16 were associated with lower school achievement in ninth‐grade standardized exams (Danish and mathematics) (Dalsgaard et al., 2020). Male students with a mental disorder had lower mean grades than their female counterparts overall, but female students diagnosed with anxiety, attention‐deficit/hyperactivity, or developmental disorders performed worse than their male counterparts (Dalsgaard et al., 2020). Second, a Norwegian registry study found that only certain mental disorders, including intellectual disabilities, personality disorders, and suicide attempts, diagnosed between ages 11 and 15 were linked to poorer school achievement by the end of compulsory education (Nordmo et al., 2022). The negative associations between these diagnoses and school achievement were slightly stronger among female students (Nordmo et al., 2022). These mixed findings highlight the need for further population‐based research to clarify the role of specific mental disorders in shaping school achievement and to better understand gender differences. It is also noteworthy that the Danish study relied solely on standardized exam scores, while the Norwegian study excluded early‐onset diagnoses before age 11. Addressing these gaps, the current study used summative grades from the final three years of comprehensive school to better reflect overall school achievement, and included diagnoses from age 1 through graduation, thereby capturing both early‐ and late‐onset disorders.

Furthermore, the role of age at onset has not been examined in detail in previous studies. Mental disorders typically emerge in childhood or adolescence (Kessler et al., 2007), with a peak onset around age 14.5 (Solmi et al., 2022). Traditional childhood‐onset disorders, such as intellectual disabilities and developmental disorders, generally have the earliest onset (McGrath, Al‐Hamzawi, Alonso, et al., 2023; Solmi et al., 2022). While prior investigations have established associations between early‐onset mental disorders and poorer school achievement during adolescence (Breslau et al., 2009; Leach & Butterworth, 2012), the differential impact of disorder onset age remains underexplored. A meta‐analysis found no overall differences in school achievement by age at onset (Riglin, Petrides, Frederickson, & Rice, 2014), but findings differ in studies that assess specific time points of mental disorder onset. For example, a longitudinal study found externalizing problems at age 3 predicted school achievement at age 12 (Agnafors, Barmark, & Sydsjö, 2021), while another study found externalizing and internalizing problems at age 6 were not associated with academic achievement at age 17 (Breslau et al., 2009). These inconsistencies suggest that the relationship between mental disorder and school achievement may depend on the disorder type and the specific timing of onset. A comprehensive understanding of how age at onset influences school achievement remains lacking.

This population‐based nationwide cohort study examined the association between childhood mental disorders and subsequent school achievement at the end of comprehensive education and whether these associations differed between (a) male and female students, and (b) age at onset of mental disorders. First, we hypothesized that mental disorders diagnosed during childhood or adolescence would be associated with lower school achievement. Second, we did not formulate a hypothesis regarding gender differences due to inconsistent findings in prior research. Third, given the limited existing evidence, we did not propose a specific hypothesis concerning the role of age at onset in school achievement.

## Methods

### Data sources

The study population was constructed by integrating individual‐level information from national registers. In Finland, everyone is assigned a unique pseudonymized identification number, which can be used to link information from different registers. Annual socio‐demographic information was retrieved from the Finnish Population Register (FOLK) of Statistics Finland, which is available for all residents since 1987.

Mental disorder diagnoses were retrieved from the Hospital Discharge Register (HDR), the Care Register for Health Care (CRHC), and the Register of Primary Healthcare Visits (RPHV) maintained by the Finnish Institute of Health and Welfare. The HDR includes secondary inpatient health care visits in 1970–1993; the CRHC includes secondary inpatient visits since 1994 and outpatient visits since 1998; and the RPHV includes primary health care visits since 2011.

In the Finnish school system, comprehensive education begins in the year children turn seven. The system comprises primary education (grades 1–6) and lower secondary education (grades 7–9) and is uniform, with no academic or vocational tracking. Over 95% of children attend publicly funded schools, including the small proportion enrolled in regulated private schools, all of which are covered by national education registers. School achievement data were obtained from the National Joint Application Register, which includes the comprehensive school grades of students who applied to secondary education between 1996 and 2019. The register covers both public and private schools. In Finland, children with special educational needs are educated in mainstream schools under an inclusive education system. Special schools and special classes within mainstream schools account for only a small proportion of students (Statistics Finland, 2025) and are included in national registers when GPA data are available.

According to Finnish law, informed consent is not required for register‐based studies.

### Study population

The study population consisted of individuals born between January 1, 1990, and December 31, 2003, who completed their comprehensive education in Finland during the years 2005 to 2019 (see Figure 1 for details). Individuals who were schooled abroad, died before they graduated, or whose school achievement information was missing from the registers were excluded from the study (*n* = 158,783). In Finland, students generally complete their comprehensive education in the year they reach 16 years of age. We additionally included individuals who graduated in the year they turned 15 or 17, while excluding those who completed their studies at any other ages (*n* = 7,715).

**Figure 1:**
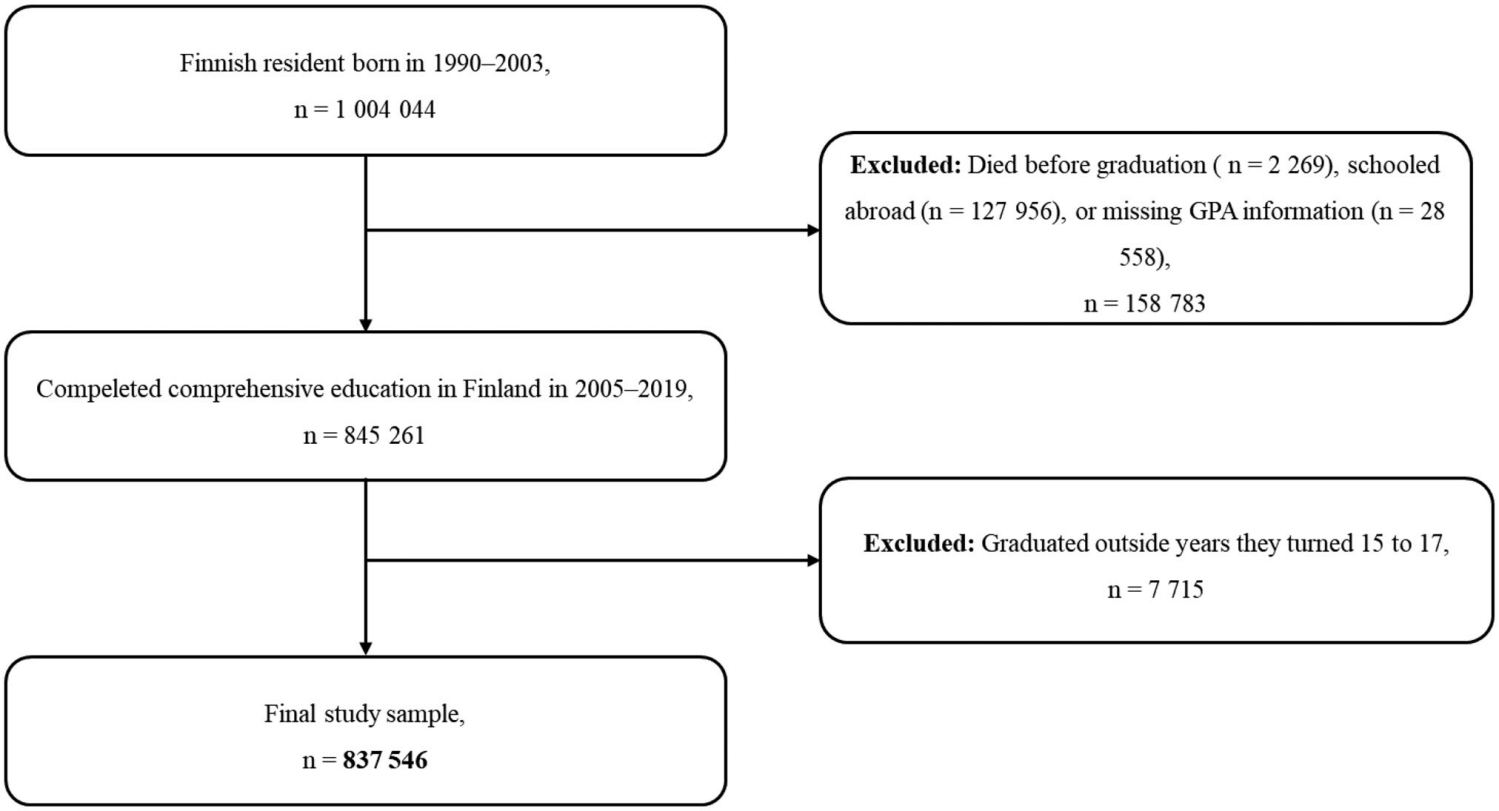
Flowchart of sample selection for the study cohort

### Measures

#### Exposures

Exposure to any mental disorder was defined using the subchapter F of the International Statistical Classification of Diseases and Related Health Problems 10th Revision (ICD‐10; F00–F99). ICD‐9 codes (used in 1987–1995) and the International Classification of Primary Care, 2nd edition (ICPC‐2) codes (used by some primary care providers) were converted to the corresponding ICD‐10 codes (used since 1996) (see Table S1). We included diagnoses recorded between the age of 1 year and May 31 in the year of graduation from comprehensive school.

Moreover, we examined exposure to specific mental disorders separately. Specific disorders were classified into the following subchapter F categories of the ICD‐10: substance use disorders (F10–F19), psychotic disorders (F20–F29), mood disorders (F30–F39), anxiety disorders (F40–F48), eating disorders (F50), intellectual disabilities (F70–F79), developmental disorders (F84), and childhood‐onset disorders (F90–F98). For diagnostic categories with typically later onset (F10–F50), diagnoses were included from the age of 5 years onwards.

Mental disorder status was used as a binary exposure variable (0 = no mental disorder diagnosis, 1 = at least one mental disorder diagnosis) in the models. In addition, we conducted analyses with a secondary exposure, where mental disorder status was categorized according to the age at which the diagnosis was first received (0 = no mental disorder diagnosis, 1 = first mental disorder diagnosis at 1–4 years old, 2 = first mental disorder diagnosis at 5–8 years old, 3 = first mental disorder diagnosis at 9–12 years old, 4 = first mental disorder diagnosis at 13–17 years old).

#### Outcome

In Finland, most students start their comprehensive education in the year they turn 7 years old and complete it in the year they turn 16 years old. Education is offered free of charge and teaching follows a national curriculum.

School achievement was measured as a grade point average (GPA), mean of all school grades, at the end of comprehensive school. In the Finnish school system, grades are assessed on a scale of 4–10, where 4 is the lowest and 10 is the highest. The final grade given at the end of comprehensive school reflects students’ performance during the final three years of education and is assessed according to national guidelines. GPA includes all compulsory school subjects: mother tongue and literature, the other national official language, foreign languages, mathematics, physics, chemistry, biology, geography, religion/ethics, history, social studies, health studies, physical education, crafts, music, home economics, visual arts, and any optional subjects students selected. GPA is used as a selection criterion when students apply to upper secondary education, such as general upper secondary education or vocational education and training. For the years 2005–2007, the GPA was available only for individuals who applied to an upper secondary education. From 2008 to 2019, the information was available for all individuals who completed their comprehensive education.

The GPA was standardized into a *z*‐score by subtracting the sample mean and dividing by the sample standard deviation. Standardization was conducted separately for each graduation year to account for variations in grading and data collection over time.

#### Confounders

The following confounders were included based on previous literature (Bradley & Corwyn, 2002; Peen, Schoevers, Beekman, & Dekker, 2010; Rutter, Caspi, & Moffitt, 2003) and the availability of data in national registers: gender which is obtained from the Finnish Population Register and reflects legally registered gender as of July 2025, calendar year of graduation which was treated as a categorical variable, the level of urbanization in the residence in the birth year (0 = missing, 1 = urban, 2 = semi‐urban, 3 = rural), parental age at birth (0 = missing, 1 = ≤25 years old, 2 = 26–30 years old, 3 = 31–35 years old, 4 = ≥36 years old), and parental education at the child's birth (0 = missing, 1 = lower secondary or less, 2 = upper secondary, 3 = post‐secondary or tertiary).

### Statistical analysis

We employed linear regression models to evaluate the association of childhood mental disorders with GPA at graduation from comprehensive school. The estimated differences in standardized GPA by mental disorder diagnosis, along with their 95% confidence intervals, were interpreted as differences measured in standard deviations.

To analyze potential differences by age at onset, we implemented additional linear regression models where the exposure was categorized according to the age at first mental disorder diagnosis. All analyses were performed both for the total population, as well as for each gender separately, and adjusted for calendar year of graduation, the urbanization in the residence in the birth year, parental age at birth, parental education at child's birth, and gender (for total population only).

Additionally, we conducted a comorbidity analysis to examine whether mental disorder comorbidity was associated with school achievement by categorizing exposure according to the number of diagnostic categories recorded before completion of comprehensive school (0, 1, 2, or ≥3). We also repeated the analyses after excluding intellectual disabilities and developmental disorders (ICD‐10 codes F70–F79 and F84).

Data were analyzed in Stata version 17.0 (StataCorp) and R version 4.2.2 (The R Foundation) using the packages tidyverse (Wickham et al., 2019) and gtsummary (Sjoberg, Whiting, Curry, Lavery, & Larmarange, 2021).

## Results

Table 1 presents the descriptive information of the study population. Between age 1 and graduation (the year one turns 16), 16.41% of students were diagnosed with at least one type of mental disorder.

**Table 1:** Descriptive information of the study population

Characteristics	Total (*N* = 837,546)	Female (*N* = 411,054)	Male (*N* = 426,492)
	Median (IQR)	Median (IQR)	Median (IQR)
Birth year	1996.0 (1993.0, 2000.0)	1996.0 (1993.0, 2000.0)	1996.0 (1993.0, 2000.0)
Graduation year	2012.0 (2009.0, 2016.0)	2012.0 (2009.0, 2016.0)	2012.0 (2009.0, 2016.0)
GPA (4–10 scale)	7.9 (7.2, 8.6)	8.3 (7.6, 8.9)	7.6 (6.9, 8.3)
	***N* (%)**	***N* (%)**	***N* (%)**
Urbanization of residence			
Urban	463,452 (55.33%)	227,376 (55.32%)	236,076 (55.35%)
Semi‐urban	135,856 (16.22%)	66,903 (16.28%)	68,953 (16.17%)
Rural	207,294 (24.75%)	101,414 (24.67%)	105,880 (24.83%)
Missing	30,944 (3.69%)	15,361 (3.74%)	15,583 (3.65%)
Mother's education			
Lower secondary or less	133,795 (15.97%)	65,593 (15.96%)	68,202 (15.99%)
Upper secondary	348,147 (41.57%)	171,085 (41.62%)	177,062 (41.52%)
Post‐secondary or tertiary	327,918 (39.15%)	160,638 (39.08%)	167,280 (39.22%)
Missing	27,686 (3.31%)	13,738 (3.34%)	13,948 (3.27%)
Father's education			
Lower secondary or less	162,841 (19.44%)	80,515 (19.59%)	82,326 (19.30%)
Upper secondary	385,795 (46.06%)	189,029 (45.99%)	196,766 (46.14%)
Post‐secondary or tertiary	251,184 (29.99%)	122,808 (29.88%)	128,376 (30.10%)
Missing	37,726 (4.50%)	18,702 (4.55%)	19,024 (4.46%)
Mother's age			
−25	214,717 (25.64%)	105,197 (25.59%)	109,520 (25.68%)
26–30	295,514 (35.28%)	144,990 (35.27%)	150,524 (35.29%)
31–35	221,181 (26.41%)	108,590 (26.42%)	112,591 (26.40%)
36–	102,631 (12.25%)	50,513 (12.29%)	52,118 (12.22%)
Missing	3,503 (0.42%)	1764 (0.43%)	1739 (0.41%)
Father's age			
−25	118,243 (14.12%)	57,662 (14.03%)	60,581 (14.20%)
26–30	256,714 (30.65%)	125,847 (30.62%)	130,867 (30.68%)
31–35	251,054 (29.97%)	123,402 (30.02%)	127,652 (29.93%)
36‐	192,560 (22.99%)	94,535 (23.00%)	98,025 (22.98%)
Missing	18,975 (2.27%)	9,608 (2.34%)	9,367 (2.20%)
Mental disorder diagnosis			
Substance use disorders	6,538 (0.78%)	3,146 (0.77%)	3,392 (0.80%)
Psychotic disorders	1873 (0.22%)	1,040 (0.25%)	833 (0.20%)
Mood disorders	24,787 (2.96%)	16,573 (4.03%)	8,214 (1.93%)
Anxiety disorders	33,763 (4.03%)	21,981 (5.35%)	11,782 (2.76%)
Eating disorders	6,171 (0.74%)	5,404 (1.31%)	767 (0.18%)
Intellectual disabilities	2,913 (0.35%)	1,138 (0.28%)	1775 (0.42%)
Developmental disorders	7,157 (0.85%)	1,596 (0.39%)	5,561 (1.30%)
Childhood‐onset disorders	68,030 (8.12%)	25,718 (6.26%)	42,312 (9.92%)
Any mental disorder	137,427 (16.41%)	61,334 (14.92%)	76,093 (17.84%)
Age at substance use disorders			
5–8 years old	19 (0.29%)	3 (0.10%)	16 (0.47%)
9–12 years old	329 (5.03%)	96 (3.05%)	233 (6.87%)
13–17 years old	6,190 (94.68%)	3,047 (96.85%)	3,143 (92.66%)
Age at psychotic disorders			
5–8 years old	59 (3.15%)	18 (1.73%)	41 (4.92%)
9–12 years old	332 (17.73%)	112 (10.77%)	220 (26.41%)
13–17 years old	1,482 (79.12%)	910 (87.50%)	572 (68.67%)
Age at mood disorders			
5–8 years old	660 (2.66%)	178 (1.07%)	482 (5.87%)
9–12 years old	3,987 (16.09%)	1,445 (8.72%)	2,542 (30.95%)
13–17 years old	20,140 (81.25%)	14,950 (90.21%)	5,190 (63.18%)
Age at anxiety disorders			
5–8 years old	2,333 (6.91%)	958 (4.36%)	1,375 (11.67%)
9–12 years old	6,289 (18.63%)	3,010 (13.69%)	3,279 (27.83%)
13–17 years old	25,141 (74.46%)	18,013 (81.95%)	7,128 (60.50%)
Age at eating disorders			
5–8 years old	159 (2.58%)	91 (1.68%)	68 (8.87%)
9–12 years old	1,021 (16.55%)	779 (14.42%)	242 (31.55%)
13–17 years old	4,991 (80.88%)	4,534 (83.90%)	457 (59.58%)
Age at intellectual disabilities			
1–4 years old	248 (8.51%)	92 (8.08%)	156 (8.79%)
5–8 years old	821 (28.18%)	283 (24.87%)	538 (30.31%)
9–12 years old	655 (22.49%)	273 (23.99%)	382 (21.52%)
13–17 years old	1,189 (40.82%)	490 (43.06%)	699 (39.38%)
Age at developmental disorders			
1–4 years old	637 (8.90%)	163 (10.21%)	474 (8.52%)
5–8 years old	2035 (28.43%)	357 (22.37%)	1,678 (30.17%)
9–12 years old	2,434 (34.01%)	483 (30.26%)	1951 (35.08%)
13–17 years old	2051 (28.66%)	593 (37.16%)	1,458 (26.22%)
Age at childhood‐onset disorders			
1–4 years old	2,776 (4.08%)	1,111 (4.32%)	1,665 (3.94%)
5–8 years old	19,456 (28.60%)	5,850 (22.75%)	13,606 (32.16%)
9–12 years old	22,733 (33.42%)	6,781 (26.37%)	15,952 (37.70%)
13–17 years old	23,065 (33.90%)	11,976 (46.57%)	11,089 (26.21%)
Age at any mental disorder			
1–4 years old	11,594 (8.44%)	3,486 (5.68%)	8,108 (10.66%)
5–8 years old	33,287 (24.22%)	9,705 (15.82%)	23,582 (30.99%)
9–12 years old	33,729 (24.54%)	11,597 (18.91%)	22,132 (29.09%)
13–17 years old	58,817 (42.80%)	36,546 (59.59%)	22,271 (29.27%)

Students with a mental disorder had lower comprehensive school achievement than those without, with a mean difference of −0.41 [95% CI, −0.41 to −0.40], as shown in Figure 2. This pattern was consistent across all mental disorders except for eating disorders, which were associated with slightly higher school achievement compared with students without a diagnosis, 0.06 [95% CI, 0.04–0.08]. The largest school achievement difference was observed for substance use disorders, −0.74 [95% CI, −0.76 to −0.72], while the smallest was seen in those with eating disorders, 0.06 [95% CI, 0.04–0.08].

**Figure 2:**
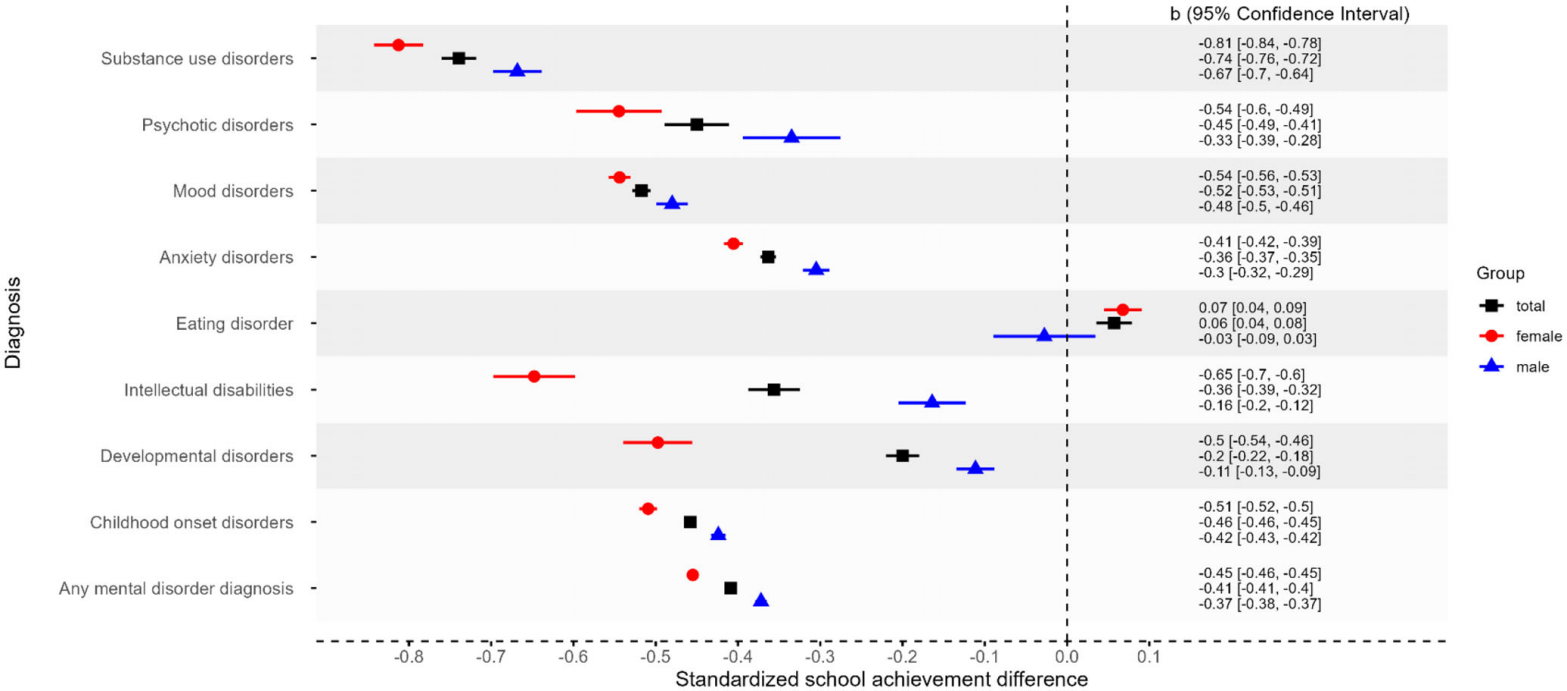
Standardized school achievement (GPA) difference at the graduation from comprehensive school between individuals with and without mental disorders

Additionally, the association between having at least one mental disorder diagnosis and school achievement was more pronounced in females than in males (female: −0.45 [95% CI, −0.46 to −0.45] vs. male: −0.37 [95% CI, −0.38 to −0.37]). The greatest gender difference was observed in intellectual disabilities (females: −0.65 [95% CI, −0.70 to −0.60] vs. males: −0.16 [95% CI, −0.20 to −0.12]), whereas the smallest difference occurred in mood disorders (females: −0.54 [95% CI, −0.56 to −0.53] vs. males: −0.48 [95% CI, −0.50 to −0.46]).

As shown in Figure 3, the later the onset of a mental disorder, the lower the overall school achievement, with the greatest association observed in those diagnosed at ages 13–17, −0.53 [95% CI, −0.54 to −0.52], followed by ages 9–12, −0.43 [95% CI, −0.44 to −0.42], ages 5–8, −0.26 [95% CI, −0.27 to −0.25], and ages 1–4, −0.17 [95% CI, −0.18 to −0.15]. This age‐related pattern was observed for substance use disorders, mood disorders, intellectual disabilities, developmental disorders, and childhood‐onset disorders (see Table 2 for numerical estimates). Furthermore, both females and males showed a clear age‐related pattern (i.e., greater association in older age groups) in terms of substance use disorders, mood disorders, intellectual disabilities, childhood‐onset disorders, and any mental disorder diagnosis. However, for anxiety disorders, there was only a clear age‐related pattern for males but not for females.

**Figure 3:**
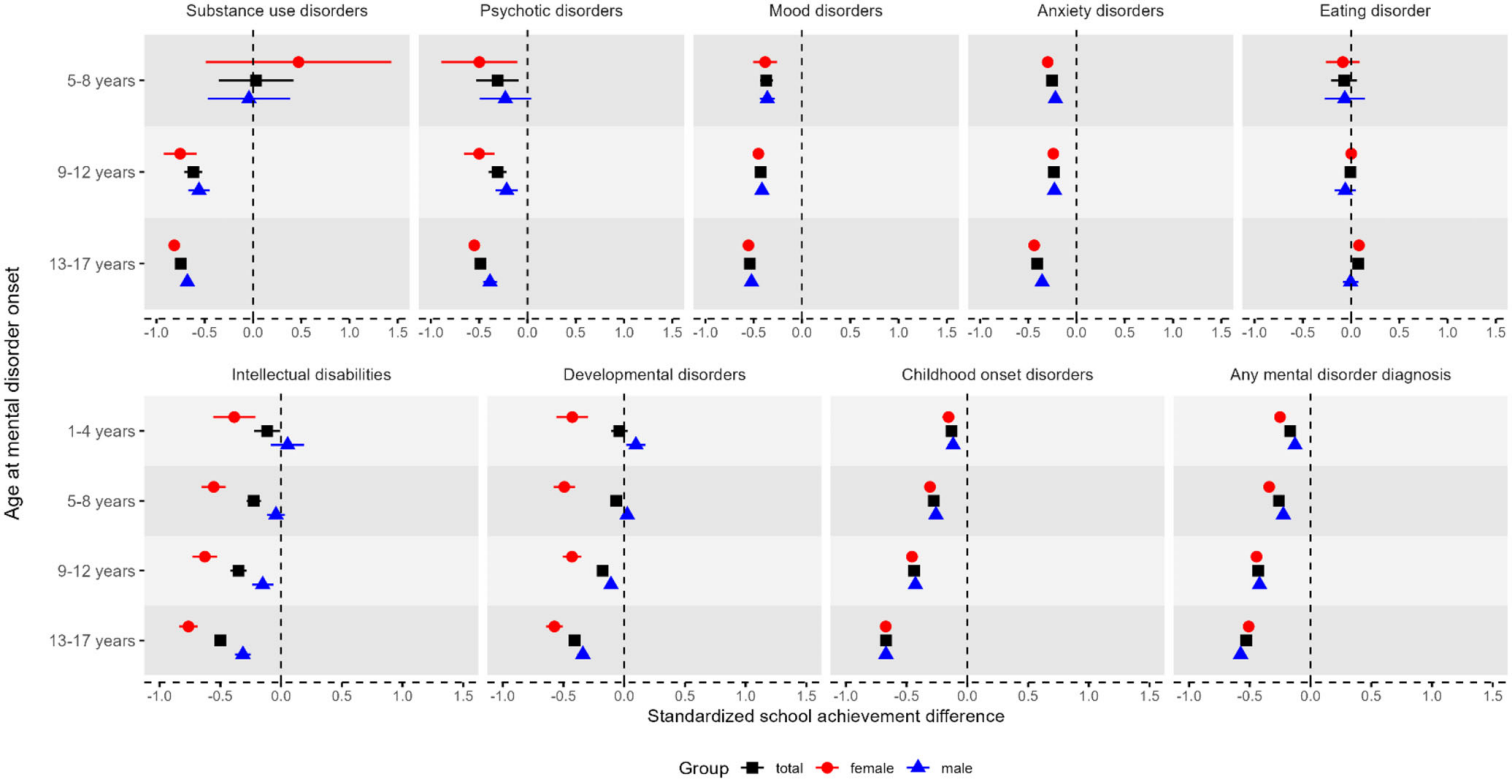
Standardized school achievement (GPA) difference by the age at onset at graduation from comprehensive school between individuals with and without mental disorders

**Table 2:** Standardized school achievement (GPA) difference by age at onset at the graduation from comprehensive school between individuals with and without mental disorders

Diagnosis	Age at mental disorder onset	Total	Female	Male
		*b* (95% CI)	*b* (95% CI)	*b* (95% CI)
Substance use disorders	No mental disorder	Ref.	Ref.	Ref.
	5–8 years	0.03 (−0.36, 0.42)	0.47 (−0.49, 1.43)	−0.04 (−0.47, 0.39)
	9–12 years	−0.62 (−0.71, −0.53)	−0.75 (−0.92, −0.58)	−0.56 (−0.67, −0.45)
	13–17 years	−0.75 (−0.77, −0.73)	−0.82 (−0.85, −0.79)	−0.68 (−0.71, −0.65)
Psychotic disorders	No mental disorder	Ref.	Ref.	Ref.
	5–8 years	−0.31 (−0.53, −0.09)	−0.50 (−0.89, −0.11)	−0.23 (−0.50, 0.04)
	9–12 years	−0.31 (−0.40, −0.22)	−0.50 (−0.66, −0.34)	−0.22 (−0.33, −0.10)
	13–17 years	−0.49 (−0.53, −0.44)	−0.55 (−0.61, −0.50)	−0.39 (−0.46, −0.32)
Mood disorders	No mental disorder	Ref.	Ref.	Ref.
	5–8 years	−0.37 (−0.43, −0.30)	−0.38 (−0.51, −0.26)	−0.36 (−0.44, −0.28)
	9–12 years	−0.43 (−0.45, −0.40)	−0.45 (−0.50, −0.41)	−0.41 (−0.45, −0.38)
	13–17 years	−0.54 (−0.55, −0.53)	−0.55 (−0.57, −0.54)	−0.52 (−0.55, −0.50)
Anxiety disorders	No mental disorder	Ref.	Ref.	Ref.
	5–8 years	−0.25 (−0.29, −0.22)	−0.30 (−0.35, −0.24)	−0.22 (−0.27, −0.17)
	9–12 years	−0.23 (−0.25, −0.21)	−0.24 (−0.27, −0.21)	−0.23 (−0.26, −0.20)
	13–17 years	−0.41 (−0.42, −0.40)	−0.44 (−0.45, −0.43)	−0.36 (−0.38, −0.34)
Eating disorder	No mental disorder	Ref.	Ref.	Ref.
	5–8 years	−0.07 (−0.21, 0.06)	−0.09 (−0.26, 0.09)	−0.07 (−0.27, 0.14)
	9–12 years	−0.01 (−0.06, 0.05)	0.00 (−0.06, 0.06)	−0.06 (−0.17, 0.05)
	13–17 years	0.07 (0.05, 0.10)	0.08 (0.06, 0.11)	0.00 (−0.09, 0.08)
Intellectual disabilities	No mental disorder	Ref.	Ref.	Ref.
	1–4 years	−0.11 (−0.22, −0.01)	−0.39 (−0.56, −0.21)	0.05 (−0.08, 0.19)
	5–8 years	−0.22 (−0.28, −0.17)	−0.55 (−0.65, −0.45)	−0.04 (−0.12, 0.03)
	9–12 years	−0.35 (−0.42, −0.28)	−0.63 (−0.73, −0.53)	−0.15 (−0.24, −0.06)
	13–17 years	−0.50 (−0.55, −0.45)	−0.76 (−0.84, −0.69)	−0.31 (−0.38, −0.25)
Developmental disorders	No mental disorder	Ref.	Ref.	Ref.
	1–4 years	−0.04 (−0.11, 0.03)	−0.43 (−0.56, −0.30)	0.10 (0.02, 0.18)
	5–8 years	−0.07 (−0.10, −0.03)	−0.49 (−0.58, −0.40)	0.03 (−0.02, 0.07)
	9–12 years	−0.18 (−0.21, −0.14)	−0.43 (−0.51, −0.35)	−0.11 (−0.15, −0.07)
	13–17 years	−0.41 (−0.45, −0.37)	−0.57 (−0.64, −0.51)	−0.34 (−0.38, −0.30)
Childhood‐onset disorders	No mental disorder	Ref.	Ref.	Ref.
	1–4 years	−0.13 (−0.16, −0.10)	−0.15 (−0.20, −0.11)	−0.12 (−0.16, −0.08)
	5–8 years	−0.28 (−0.29, −0.26)	−0.31 (−0.33, −0.29)	−0.26 (−0.27, −0.24)
	9–12 years	−0.44 (−0.45, −0.43)	−0.46 (−0.48, −0.44)	−0.43 (−0.44, −0.41)
	13–17 years	−0.67 (−0.68, −0.66)	−0.67 (−0.69, −0.66)	−0.67 (−0.69, −0.65)
Any mental disorder	No mental disorder	Ref.	Ref.	Ref.
	1–4 years	−0.17 (−0.18, −0.15)	−0.25 (−0.28, −0.22)	−0.13 (−0.15, −0.11)
	5–8 years	−0.26 (−0.27, −0.25)	−0.34 (−0.36, −0.32)	−0.22 (−0.24, −0.21)
	9–12 years	−0.43 (−0.44, −0.42)	−0.44 (−0.46, −0.43)	−0.42 (−0.43, −0.41)
	13–17 years	−0.53 (−0.54, −0.52)	−0.51 (−0.52, −0.50)	−0.58 (−0.59, −0.56)

In the comorbidity analysis, students with diagnoses in multiple diagnostic categories showed progressively lower GPA compared with those without any diagnosis (−0.38 [95% CI, −0.39 to −0.37] for one diagnostic category, −0.50 [95% CI, −0.51 to −0.49] for two, and − 0.62 [95% CI, −0.64 to −0.60] for three or more) (see Table 3). The pattern was similar in both genders. In addition, females with diagnoses in multiple categories experienced a greater decrease in school achievement than males (−0.54 [95% CI, −0.56 to −0.53] vs. −0.53 [95% CI, −0.54 to −0.51] for two categories, and − 0.68 [95% CI, −0.71 to −0.66] vs. −0.60 [95% CI, −0.63 to −0.56] for three or more categories).

**Table 3:** Associations between mental disorder comorbidity and comprehensive school GPA

Diagnosis	Comorbidity	Total (*N* = 837,546)	Female (*N* = 411,054)	Male (*N* = 426,492)
		*b* (95% CI)	*b* (95% CI)	*b* (95% CI)
All diagnoses	No mental disorder	Ref.	Ref.	Ref.
	1	−0.38 (−0.39, −0.37)	−0.38 (−0.39, −0.37)	−0.38 (−0.39, −0.37)
	2	−0.50 (−0.51, −0.49)	−0.54 (−0.56, −0.53)	−0.45 (−0.47, −0.43)
	3	−0.62 (−0.64, −0.60)	−0.69 (−0.71, −0.67)	−0.52 (−0.55, −0.49)
Excluding intellectual disabilities and developmental disorders	No mental disorder	Ref.	Ref.	Ref.
	1	−0.38 (−0.39, −0.38)	−0.38 (−0.39, −0.37)	−0.39 (−0.39, −0.38)
	2	−0.53 (−0.54, −0.52)	−0.54 (−0.56, −0.53)	−0.53 (−0.54, −0.51)
	3	−0.65 (−0.67, −0.63)	−0.68 (−0.71, −0.66)	−0.60 (−0.63, −0.56)

Estimates represent standardized mean differences in GPA compared with students without any diagnosis. Comorbidity was defined as the number of diagnostic categories (1, 2, or ≥3). In total, 76,439 had one category within these categories, 20,821 had two, and 7,272 had three or more categories. *Excluding intellectual disabilities and developmental disorders* indicates analyses in which intellectual disabilities and developmental disorders were excluded (ICD‐10 codes F70–F79 and F84).

## Discussion

This nationwide cohort study on comprehensive school achievement in children and adolescents with and without mental disorders yielded four main findings. First, all mental disorders, diagnosed in childhood or adolescence, except for eating disorders, were associated with significantly lower comprehensive school achievement. Second, the association between mental disorders and school achievement was more pronounced in females than in males. Third, the later the onset of a mental disorder, the greater was its association with school achievement. Fourth, higher mental disorder comorbidity was associated with lower GPA.

We found that students with a mental disorder had lower overall school achievement than those without, across nearly all diagnostic categories, except for eating disorders. The associations were most notable for substance use disorders. These findings align with previous studies focusing on single mental disorders (Esch et al., 2014; Galéra et al., 2009; Keen, Webster, & Ridley, 2016; Maurizi et al., 2013) as well as studies examining multiple mental disorders (Dalsgaard et al., 2020; Nordmo et al., 2022). As reported in earlier studies, substance use has been associated with executive functioning deficits, including difficulties with attention, working memory, and both verbal and nonverbal processing (Jovanovski, Erb, & Zakzanis, 2005; Kalechstein, De La Garza, Mahoney, Fantegrossi, & Newton, 2007), all of which are important for academic success. Interestingly, students diagnosed with eating disorders demonstrated higher school achievement compared with peers without a diagnosis, a finding also supported by a previous study (Dalsgaard et al., 2020). Eating disorders have been linked to high levels of perfectionism (Bulik et al., 2003; Disorder O compulsive et al., 2014), a trait often associated with better academic performance (Bills, Greene, Stackpole, & Egan, 2023; Madigan, 2019). Our findings regarding comorbidity are also in line with previous studies showing that psychiatric comorbidity has been associated with worse prognosis and outcomes than a single diagnosis (Copeland, Wolke, Shanahan, & Costello, 2015; Plana‐Ripoll et al., 2020).

A notable gender difference emerged in the association between mental disorders and school achievement, with female students showing stronger associations than males, overall and across each diagnosis. Our findings differ from those reported in the Danish study on the overall gender difference (Dalsgaard et al., 2020), which may be because we used different measures of academic achievement. However, it aligns with previous research suggesting that female students are more academically vulnerable to intellectual disabilities, anxiety disorders, attention‐deficit hyperactivity disorders, and other developmental disorders (Dalsgaard et al., 2020; Nordmo et al., 2022). The present study extends this pattern to other diagnoses, including substance use disorders, psychotic disorders, and mood disorders. Several mechanisms may account for the heightened academic vulnerability among girls. First, female students are more sensitive to academic pressure, which can amplify the cognitive and emotional burden of mental disorders (Montolio & Taberner, 2021). Second, it may partly reflect a floor effect in male students, whose lower baseline performance, particularly with early‐onset disorders, may limit observable declines (Elwood & Murphy, 2002). Although female students generally had slightly higher average GPA than males in our data, the stronger associations observed among females are unlikely to simply reflect these baseline differences as we used standardized mean differences. Rather, the results suggest that mental disorders may be linked to greater shifts in academic performance among female students. Third, female students with mental disorders are often perceived as less impaired than males, causing their difficulties to be overlooked or underestimated (Sellers, Maughan, Pickles, Thapar, & Collishaw, 2015). Consequently, they may receive less academic or psychological support despite substantial impairments, underscoring the need for greater awareness and targeted interventions. In addition, analyses of comorbidity also indicated that females with diagnoses in multiple categories experienced a greater decrease in school achievement than males. This finding suggests that greater diagnostic complexity may partly contribute to the stronger associations observed among females.

To our knowledge, this is the first nationwide cohort study to examine the association between age at onset of mental disorders and comprehensive school achievement. We found that the association between mental disorders and school achievement was more pronounced among students with later‐onset disorders, especially during adolescence, across diagnostic categories. Several mechanisms may help explain this pattern. First, earlier identification of symptoms and sustained intervention may buffer the long‐term academic consequences of early‐onset disorders. Second, later‐onset disorders may emerge concurrently with increased academic demands and developmental transitions, potentially amplifying their disruptive impact on school performance. Notably, the adolescent period—marked by critical maturation of prefrontal cortical functions essential for executive control (Konrad, Firk, & Uhlhaas, 2013; Page & Coutellier, 2018)—may render youth particularly vulnerable to new‐onset psychopathology during this time. Third, hospital inpatient care may also contribute to lower school achievement by disrupting schooling through absences and difficulties maintaining academic engagement, although hospital schooling is commonly provided in Finland. This may be particularly relevant when hospitalization occurs closer to the end of compulsory education, potentially contributing to the larger associations observed for later‐diagnosed disorders. Fourth, students diagnosed at younger ages and attending special schools or special classes are likely to follow an individual learning plan, which could partly contribute to the observed age‐related patterns, particularly for intellectual disabilities and developmental disorders. However, as only a small proportion of students are educated in such settings (Statistics Finland, 2025), the overall impact on the findings is likely to be limited. Although registry studies have often used age at diagnosis as a proxy for age at onset (Suokas, Niemi, Gutvilig, et al., 2024), it is also important to consider that our measure reflects age at diagnosis rather than true age of symptom onset. Some students diagnosed later may have experienced prolonged periods of unrecognized or unsupported difficulties, leading to cumulative academic disadvantage by the time of diagnosis. These findings underscore the need to disentangle age at onset from age at diagnosis in future research and to ensure timely recognition and support for students whose symptoms may emerge—or be identified—during academically critical periods. Together, the findings highlight the importance of timely identification and intervention, not only for early‐onset disorders but also for those diagnosed later, particularly during critical academic transition periods.

### Strengths and limitations

To our knowledge, this is the first nationwide cohort study to examine the association between the age at diagnosis of mental disorders and school achievement. Key strengths of the study include the use of a total population cohort, the inclusion of the full range of mental disorder diagnoses, and linkage to longitudinal administrative data with diagnoses made by health care professionals. The Finnish health care registers are known for their good diagnostic validity (Sund, 2012), and the data covered both primary and secondary care settings.

However, there are also limitations to our study. First, the present study is observational, and our findings do not imply causality. Second, although we adjusted our models for several confounders, we cannot rule out the possibility of unmeasured or residual influences that may affect academic achievement. Third, students whose mental disorders were untreated were misclassified as having no mental disorder in our analyses, and we lacked information on symptoms, remission, and recovery. Fourth, our register data did not allow us to distinguish disorder severity or treatment intensity, such as inpatient versus outpatient care, although we have included an additional analysis of comorbidity. As a result, part of the observed associations, particularly for later‐onset disorders, may reflect greater clinical severity or increased school absence around the time of diagnosis. Fifth, students without a final comprehensive school GPA were excluded from the analytic sample. As school dropout or very low attendance may be more common among students with mental disorders, this selection could lead to conservative estimates of the associations. However, among those diagnosed with a mental disorder who were alive and residing in Finland at the end of comprehensive school, only a small proportion (9.1%) did not receive a final grade, suggesting that the impact of such selection on the results is likely limited. Sixth, as discussed above, in our data the age at onset reflects the age at first clinical diagnosis rather than the true onset of symptoms, and there may be a significant time lag between the onset of mental health problems and the point at which a formal diagnosis is recorded (Boulton et al., 2023). Finally, as our study is from a Nordic welfare state, the generalizability of our findings is partly limited.

## Conclusions

Students with mental disorders had lower school achievement by the end of comprehensive school, with the greatest disparity observed in substance use disorders. Notably, associations were stronger in females than in males across all disorders, suggesting a potential gender‐related difference in the academic impact of mental disorders. Additionally, later‐onset mental disorders were associated with a more pronounced decline in school achievement, highlighting the need for academic and psychological support for adolescents experiencing mental health challenges during critical educational years.

## Author contributions

Y.L.—Conceptualization, methodology, visualization, writing—original draft; R.N.—Conceptualization, methodology, formal analysis, data curation, writing—review and editing; M.G.—Conceptualization, methodology, formal analysis, data curation, writing—review and editing; M.E.—Conceptualization, writing—review and editing; M.F.—Conceptualization, writing—review and editing; C.H.—Conceptualization, supervision, project administration, funding acquisition, writing—review and editing.

## Ethical considerations

According to Finnish law, informed consent/assent was not required for register‐based studies; therefore, informed consent was waived. The study was approved by the Ethics Committee of the Finnish Institute of Health and Welfare (approval number THL/22/6.02.01/2019; approval date: 15 January 2019). Data linkage was authorized by Statistics Finland (TK‐53‐1696‐16) and the Finnish Institute of Health and Welfare.

**Key points**

**What's known?**

- Childhood mental disorders are associated with poorer school achievement, but large‐scale evidence remains limited, particularly regarding differences by age at onset. To our knowledge, this is the first nationwide cohort study to examine the association between age at onset of mental disorders and comprehensive school achievement.

**What's new?**

- Using nationwide register data (*n* = 837,546), we found that students with a mental disorder had lower school achievement than peers without a diagnosis, with stronger associations in females than males.
- Later‐onset mental disorders were associated with poorer school achievement.

**What's relevant?**

- School support systems for early identification and intervention might improve learning, especially for females and later‐onset cases. Future research should examine mechanisms (e.g. social exclusion) and test interventions to reduce achievement gaps.

## Supporting information


**Table S1.** Diagnostic categories of mental disorders.

## Data Availability

Data for this study are property of Finnish Institute of Health and Welfare and Statistics Finland. The data are available from these authorities, but restrictions apply, please see: www.findata.fi.
