# Supplementary material for: Mental disorders in childhood or adolescence and subsequent comprehensive school achievement
Source: J Child Psychol Psychiatry. 2026 Jun 4;67(11):1796–806. doi: 10.1111/jcpp.70181 (PMC13647093; doi:10.1111/jcpp.70181)
Supplement: Supplementary file 1 [file JCPP-67-1796-s001.docx]

**Mental Disorders in Childhood or Adolescence and Subsequent Comprehensive School Achievement**

**Supporting Information**

**Table S1.** Diagnostic Categories of Mental Disorders

| **Mental disorder category** | **ICD-10** | **ICD-9** | **ICPC-2** |
| --- | --- | --- | --- |
| Substance use disorders | F10–F19 | 291, 292, 303, 304, 305 | P15, P16, P17, P19 |
| Psychotic disorders | F20–F29 | 295, 297, 298 | P72 |
| Mood disorders | F30–F39 | 296, 311 | P73 |
| Anxiety disorders | F40–F48 | 300, 307.8, 309 | P02, P74, P75, P78, P79, P82 |
| Eating disorders | F50 | 307.1, 307.5 | P86 |
| Intellectual disabilities | F70–F79 | 317, 318, 319 | P85 |
| Developmental disorders | F84 | 299 |  |
| Childhood onset disorders | F90–F98 | 307.2, 307.3, 307.6, 307.7, 307.9, 308.0, 313, 314 | P10, P11, P12, P13, P22, P23, P81 |
| Any mental disorder | F00–F99 | All of the above + 290, 293, 294, 301, 302, 307.4, 310, 312, 315, 316 | All of the above + P06, P07, P08, P09, P18, P24, P29, P70, P71, P76, P80, P98, P99 |
